# Dual-binding nanoparticles improve the killing effect of T cells on solid tumor

**DOI:** 10.1186/s12951-022-01480-z

**Published:** 2022-06-07

**Authors:** Zhenyu Luo, Lihua Luo, Yichao Lu, Chunqi Zhu, Bing Qin, Mengshi Jiang, Xiang Li, Yingying Shi, Junlei Zhang, Yu Liu, Xinyu Shan, Hang Yin, Guannan Guan, Yongzhong Du, Ningtao Cheng, Jian You

**Affiliations:** 1 College of Pharmaceutical Sciences, Zhejiang University, 866 Yuhangtang Road, Hangzhou, 310058 Zhejiang People’s Republic of China; 2 School of Public Health, Zhejiang University School of Medicine, 866 Yuhangtang Road, Hangzhou, 310058 Zhejiang People’s Republic of China

**Keywords:** Adoptive cell therapy, Magnetic targeting, CD8 + T cell, Solid tumor

## Abstract

Adoptive cell therapy (ACT) was one of the most promising anti-tumor modalities that has been confirmed to be especially effective in treating hematological malignancies. However, the clinical efficacy of ACT on solid tumor was greatly hindered by the insufficient tumor-infiltration of cytotoxic CD8 + T cells. Herein, we constructed a nanoplatform termed dual-binding magnetic nanoparticles (DBMN) that comprised PEG-maleimide (Mal), hyaluronic acid (HA) and Fe_3_O_4_ for adoptive T cell-modification and ACT-sensitization. After a simple co-incubation, DBMN was anchored onto the cell membrane (Primary linking) via Michael addition reaction between the Mal and the sulfhydryl groups on the surface of T cells, generating magnetized T cells (DBMN-T). Directed by external magnetic field and in-structure Fe_3_O_4_, DBMN-T was recruited to solid tumor where HA bond with the highly expressed CD44 on tumor cells (Secondary Linking), facilitating the recognition and effector-killing of tumor cells. Bridging adoptive T cells with host tumor cells, our DBMN effectively boosted the anti-solid tumor efficacy of ACT in a mouse model and simultaneously reduced toxic side effects.

**Supplementary Information:**

The online version contains supplementary material available at 10.1186/s12951-022-01480-z.

## Introduction

In the past two decades, adoptive cell therapy (ACT) including the most clinically applied CAR-T, TCR modified T cells (TCR-T) and tumor-infiltrating lymphocytes (TIL) therapy, has achieved a great success in and provided new possibilities for the treatment of malignancies [1–3], especially blood system tumors [4–6].

Compared with traditional therapeutic modalities, ACT -based immunotherapy not only reduced the side effect of chemotherapy and radiotherapy, but exhibited a more powerful anti-tumor effect, especially in completely eradicating some blood system tumors [7, 8]. However, the application of ACT in solid tumors was extremely restrained by the fact that only limited T cells gathered on the tumor sites [9–11]. Most T cells were blocked by immunosuppressive microenvironment and dense tumor barrier.

To solve this problem, some advanced treatments have developed, such as photothermal therapy [12], photodynamic therapy [13], gene therapy [14] and personalized vaccine [15]. What’s more, well-designed nanoparticles also have been used to enhance the effect of against solid tumors [16, 17]. Meanwhile, ACT with a higher killing capability was also developed, like being modified with nanoparticles [18–20] (containing cytokines or immune checkpoint inhibitors (ICIs)) or using the new generation of CAR-T [21–23] (secreting cytokines or ICIs by T cells), but it did not solve the dilemma. More effective and convenient methods were urgently needed.

Some researchers learned from drugs delivery magnetic nanoparticles. The published studies showed magnetic nanoparticles could also control cells path, like erythrocytes [24], macrophages[25], NKs[26], as well as T cells [27]. Unfortunately, we found although the number of T cells (modified with conventional magnetic nanoparticles) could significantly increase in the experiments, it fell sharply as soon as the magnetic field removed, which meant patients need keep stiff in an intense magnetic field or an MRI machine for a long time and poor treatment efficiency. Thus, a linker between cytotoxic T lymphocyte (CTL) and tumor cell might enhance the residence time and killing ability of T cells [28, 29].

**Scheme 1:**
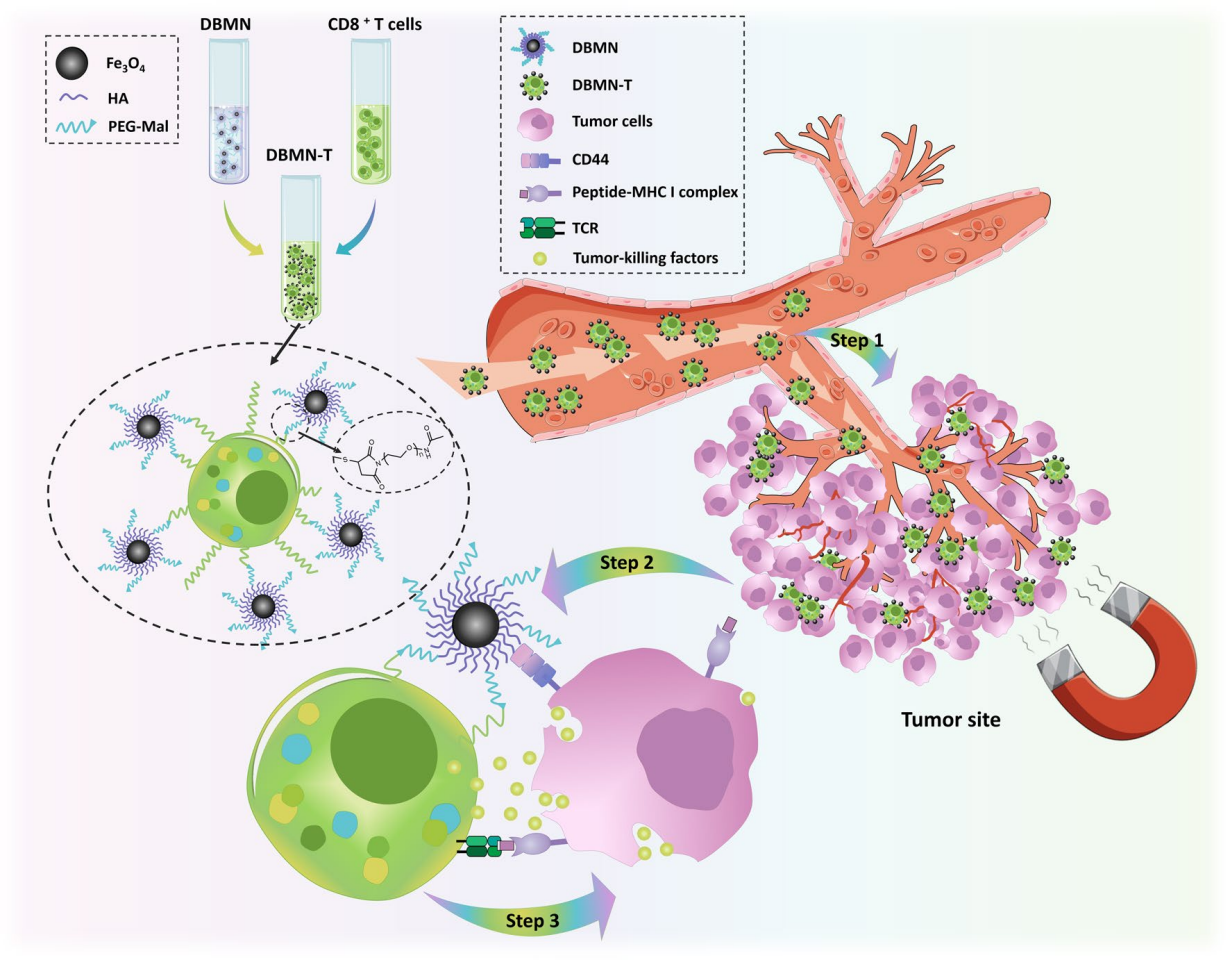
Schematic illustration of the anti-solid tumor mode of action by DBMN. DBMN-T was prepared by a simple co-incubation of DBMN and T cells in vitro, which was further used for adoptive transfer and directed to the tumor site in vivo under an external magnetic field. The HA on DBMN combined with CD44 to build a bridge between cytotoxic T cell and tumor cells, facilitating the recognition and killing to tumor cells by T cells.

In this work, dual-binding magnetic nanoparticles modified CD8 + T cells (DBMN-T) were prepared through the Michael addition reaction between the maleimide groups on the magnetic nanoparticles (Additional file 1: Fig. S1) and the free sulfhydryl groups on T cells. Compared with unmodified counterparts, our DBMN-T had a control center to accept external commands and converged to the tumor sites during blood circulation, which avoided the accidental injury of CD8 + T cells to normal tissues [30] and boosted the anti-tumor effect. As shown in Scheme 1, DBMN-T achieved first-level guidance under magnetic field and entered into the tumor sites. In the tumor microenvironment (TME), the HA on the surface of the magnetic nanoparticles provided second-level guidance, as the highly expressed CD44 molecules (the receptor for HA) on the surface of tumor cells were interacted with HA [31–33]. Thirdly, the TCR on DBMN-T specifically recognized the MHC-peptide complex on the tumor cells. By secreting cytolytic factors and cytokines such as granzyme B and perforin, DBMN-T successfully eliminated tumor cells. Although HA-mediated tumor targeting and magnetic targeting have been reported, but their combination, especially with double linking, making adoptive CD8 + T cells settle down at tumor site, greatly improved the therapeutic effect of adoptive T-cell therapy.

## Results

### Characterization of nanoparticles

HMN (HA modified magnetic nanoparticles) and DBMN were synthesized by amide reaction, with two transitions of surface charge observed: from positive (22.8 ± 2.3 mV) to negative (− 39.4 ± 3.6 mV), then to slight negative (− 5.3 ± 4.4 mV) (Additional file 1: Fig. S2A). The first transition was mainly due to the free carboxyl groups after the modification of nanoparticles with HA. The second change was resulted from the appearance of amide bonds. In the meantime, the size of the nanoparticles slightly increased from 200 ± 23 nm to 330 ± 42 nm (Additional file 1: Fig. S2B). It was reported that magnetic nanoparticles with a size of 200–300 nm possess strong magnetic properties and biological applications [34]. Additional file 1: Fig. S3A, B showed the Thermogravimetric Analysis (TGA) and Differential Scanning Calorimeter (DSC) curves of the DBMN, indicating that the HA and Mal-PEG had gradually grafted onto the surface of magnet nanoparticles, which was further verified by the completely different images of the residue after 1000 °C heating in Additional file 1: Fig. S3C. Moreover, characteristic differences were also displayed on ultraviolet-visible light absorption spectroscopy (Additional file 1: Fig. S4). All the above results confirmed the successful synthesized of DBMN. Then, changes in magnetic properties before and after modification were testified. As displayed in Additional file 1: Fig. S5, nanoparticles showed similar response to the magnetic field, proving that the modification of HA and Mal did affect their magnetic responsiveness.

**Fig. 1:**
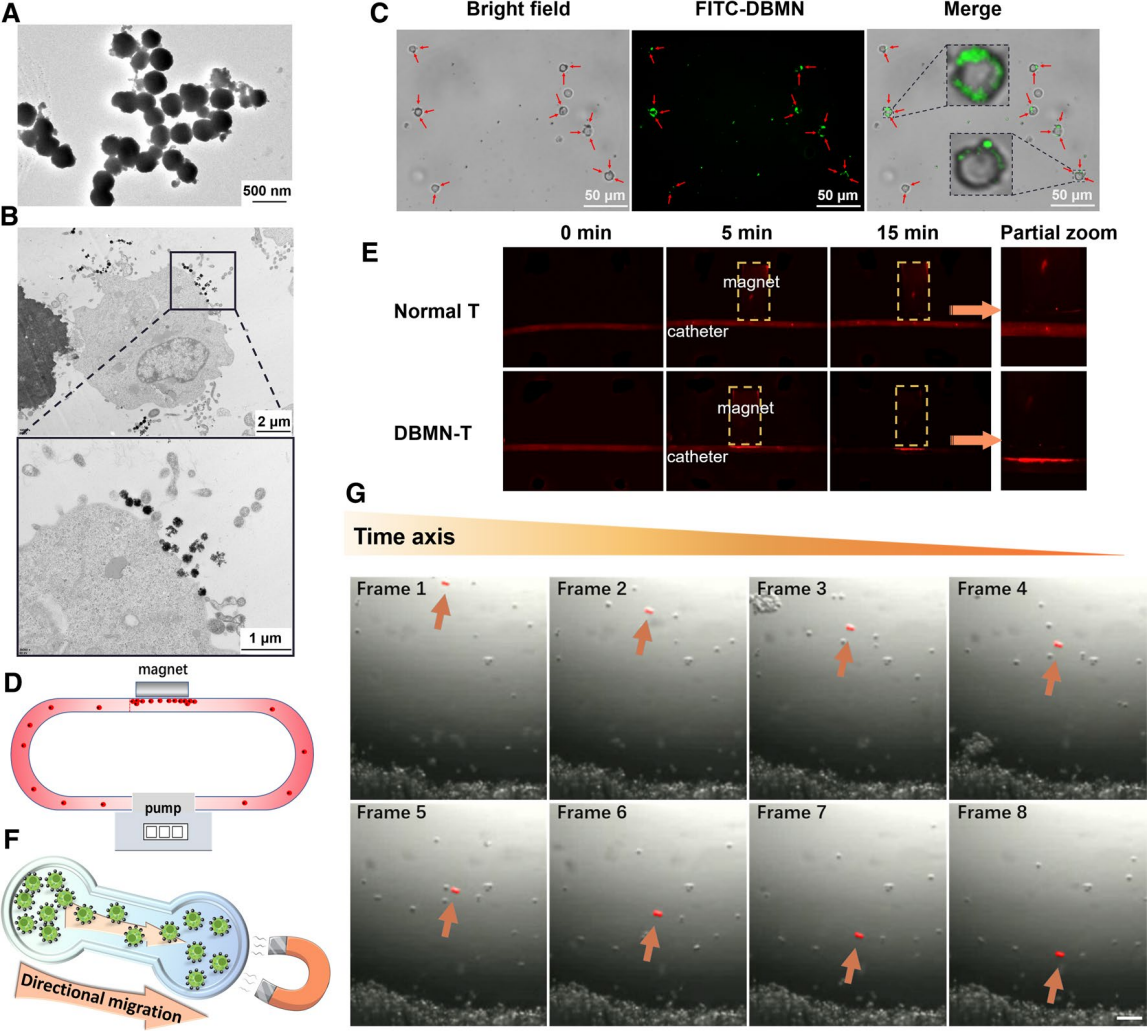
T cells combined with magnetic nanoparticles to obtain magnetic T cells. TEM images of DBMN (**A**) and T cell with nanoparticles binding on the surface (**B**). **C** Fluorescence pictures of FITC-DBMN after binding to CD8 + T cells. Green, fluorescence signal of FITC-labeled DBMN. Scale bar, 50 μm. **D** Schematic diagram of extracorporeal cell circulation under magnetic field. **E** DiD-labeled DBMN-T aggregated under an external magnetic field like (**D**) captured by IVIS system. Yellow dotted box, the location of the magnet. **F** Drop cells on one side of the culture medium and cells’ movement was observed under microscope. **G **A partial screenshot of DBMN-T moving under the magnetic field photographed by camera in microscope. Scale bar, 50 μm. The orange arrows indicate the movement of one of the cells (red) over time. See the supporting movie (Additional file 2: Movie S1) for the movement of all cells and visual fields. Normal T means unmodified CD8 + T cells; DBMN-T means DBMN engineered CD8 + T cells

We next co-incubated activated CD8 + T cells with our DBMN mainly according to references reported before [35–37]. 1 mg/mL incubation concentration of DBMN was selected according to the magnetic response rate of T cells (Additional file 1: Fig. S6). The magnetic nanoparticles modified with HA and Mal (Fig. 1A) were tightly bound on the surface of the CD8 + T cells as observed by transmission electron microscope (TEM) (Fig. 1B). Besides, the green fluorescent signal of FITC-DBMN was located on the cell surface of CD8 + T cells, coincided with the location of the nanoparticles (Fig. 1C), suggesting that the nanoparticles were indeed grafted on the cell membrane.

### The conjugation of cells and nanoparticles

Then, the magnetic responsiveness of magnetic T cells and the motion state of DBMN-T under magnetic field were verified in vitro. Firstly, DBMN-HEK293T-GFP cells were constructed and circulated in the catheter by peristaltic pump with a magnet placed on one side (Additional file 1: Fig. S7A). Results demonstrated that the strong green fluorescence was on the side close to magnet (Additional file 1: Fig. S7B), indicating that a large number of HEK293T-GFP cells gathered on this side. And the same phenomenon was observed when the device was placed into the in vivo imaging system (IVIS) (Additional file 1: Fig. S8, Fig. 1D). Then, the medium was removed and the catheter was re-perfused with PBS. After quickly rinsing 15 min, high intensity green fluorescence was still remained at the pole of the magnet, signifying that magnetic HEK293T-GFP cells were firmly adsorbed (Additional file 1: Fig. S9). Refilling the culture medium into the tube and removing the magnet simultaneously, we were delighted to find that the cells stopped aggregating, and the original aggregated cells were also scattered into medium. Next, DiD-labeled DBMN-T were used to repeat this experiment. Unsurprisingly, DBMN-T accumulated near the magnet, while normal CD8 + T cells had no tropism to magnetic field (Fig. 1E). In order to monitor the real-time movement of cells under magnetic field, DBMN-T were dropped on the opposite side of the magnet (Fig. 1F), and motion trajectory of cells were photographed. Obviously, DBMN-T moved rapidly under the magnetic field, and gathered at a high density to the side of the culture dish (Additional file 2: Movie S1, Fig. 1G). This unnatural phenomenon suggested that the DBMN really worked. In addition, when the position of the magnet changed, the DBMN-T could quickly rotate and adjust direction in the culture medium (Additional file 3: Movie S2). These evidences indicated that the response of DBMN-T to the magnetic field was remarkably sensitive and rapid.

### The function of magnetic CD8 + T cells

**Fig. 2:**
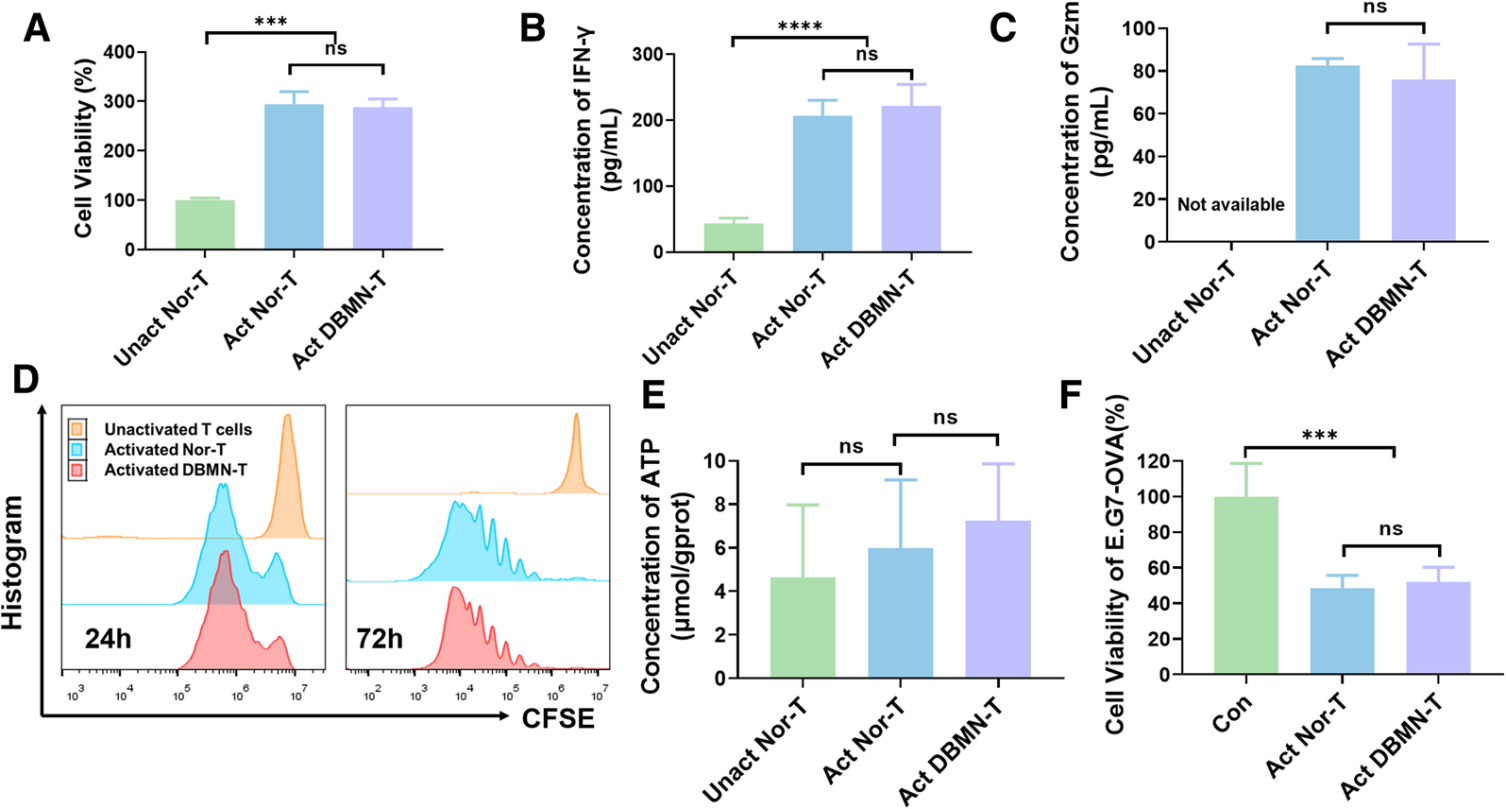
Cellular activity of DBMN-T. **A** Cell viability of DBMN-T and normal T cells after being cultured for 24 h were measured by CCK-8 kit, n = 3. The concentration of IFN-γ (**B**) and granzyme B (**C**) in the culture medium at 24 h detected by ELISA kit (OT-I T cells : E.G7-OVA = 20 : 1, n = 4). **D** the proliferation of CFSE-labeled normal CD8 + T cells and DBMN-T at 24 h (left) and 72 h (right) after activation detected by flow cytometry. **E** The amount of ATP produced by T cells or DBMN-T for 24 h, n = 3. **F** Cell viability of E.G7-OVA at 24 h detected by LDH assay kit (OT-I T cells : E.G7-OVA = 20 : 1, n = 5). Unact means unactivated; Act means activated; Nor-T means unmodified CD8 + T cells; DBMN-T means DBMN engineered CD8 + T cells. Statistical analyses were carried out by Prism graphpad 8.0. All error bars are expressed as ± SD. Comparisons between two or several groups were analyzed using unpaired Student’s t-test, *p < 0.05, **p < 0.01, ***p < 0.001

Stephan’s study [35] showed that the nanoparticles connected cell by thiols and maleimide groups could cover a limited surface area of T cells and had little effect on the function of T cells. The cytotoxicity of nanoparticles to CD8 + T cells was assayed by CCK-8 kit. The histograms showed that the nanoparticles attached to the cell had little toxicity to T cells and did not affect its ability of proliferation (Fig. 2A). And the results of Elisa assay demonstrated that there was no significant difference in the contents of IFN-γ and granzyme B in the culture medium of activated magnetic OT-I T cells (DBMN-T) and activated unmodified OT-I T cells (Nor-T) incubated with E.G7-OVA tumor cells for 24 h (Fig. 2B, C). Moreover, CFSE dilution assay showed that linking with magnetic nanoparticles did not influence the proliferation of activated T cells (Fig. 2D). As the function of T cells was closely related to energy metabolism [38], we tested the ATP level in normal CD8 + T cells and DBMN-T. The data in Fig. 2E revealed that nanoparticles did not interfere with the respiratory metabolism of T cells. LDH assay results (Fig. 2F) unsurprisingly verified the killing ability of DBMN-T, which was an important basis for further research.

### The magnetic targeting ability of DBMN-T in vivo

**Fig. 3:**
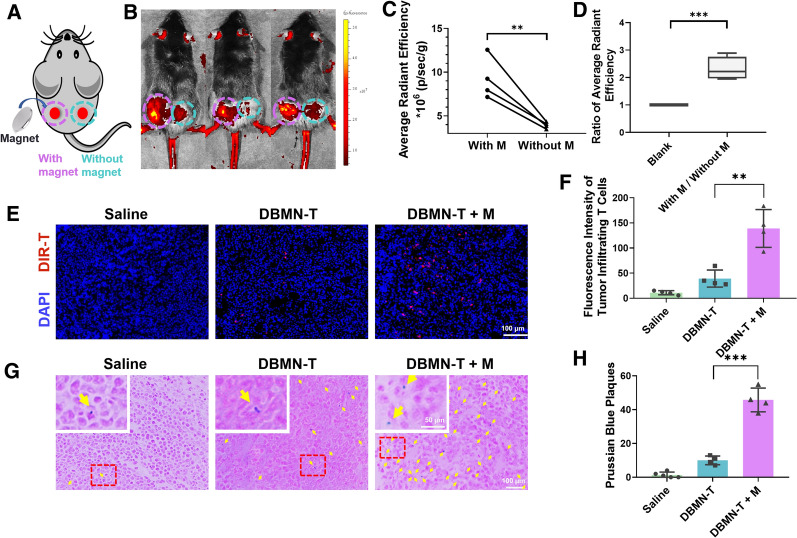
Magnetic responsiveness of DBMN-T in vivo. **A** Schematic diagram of a bilateral tumor model on the back of mouse. **B** Fluorescence images of DiR-labeled DBMN-T rearrangement in vivo under the magnetic field for 48 h photographed by IVIS system. Pink circle, under magnetic field; Blue circle, without magnetic field. **C** Fluorescence semi-quantitative result of (**B**) by software in IVIS system. **D** The ratio of the fluorescence intensity of the magnetic field to the opposite side at the same mouse, n = 4. Blank, no magnetic field effect, the default is 1. **E** Representative immunofluorescence images of labeled T cells in tumor and (**F**) is semi-quantitative fluorescence of (**E**) by Image J. **G** Representative Prussian blue staining images of tumor. Yellow arrows indicate the position of blue plaques and the number was shown in (**H**) counted by Image J. Scale bar, 100 μm. Plus M means external magnetic field. Statistical analyses were carried out by Prism graphpad 8.0. All error bars are expressed as ± SD. Comparisons between two or several groups were analyzed using unpaired Student’s t-test, *p < 0.05, **p < 0.01, ***p < 0.001, ****p < 0.0001

To investigate the targeting ability of DBMN-T in vivo, bilateral subcutaneous E.G7-OVA tumor model was established. After the tumor volume reached 500mm^3^, DiR-labeled DBMN-T were intravenously injected, and a magnet was bound to left tumor by double-sided tape (3 M) which did not affect the diet and daily activities of mice. (Fig. 3A). 48 h later, mice with two tumors were photographed by IVIS. Results show that the average fluorescence intensity of the magnet-placed side was 2.3 times higher than that of the other side (Fig. 3B–D). Then, tumors were isolated to detect the amount of labeled CD8 + T cells. In line with the expectation, the infiltration of labeled CD8 + T cells extremely increased under magnetic field (Fig. 3E, F). Furthermore, the iron oxide nanoparticles in the tissues increased accordingly (Fig. 3G, H). These data demonstrated that DBMN-T could accept external commands and march to solid tumors in vivo.

### Antitumor effect in subcutaneous tumor model and safety evaluation

**Fig. 4:**
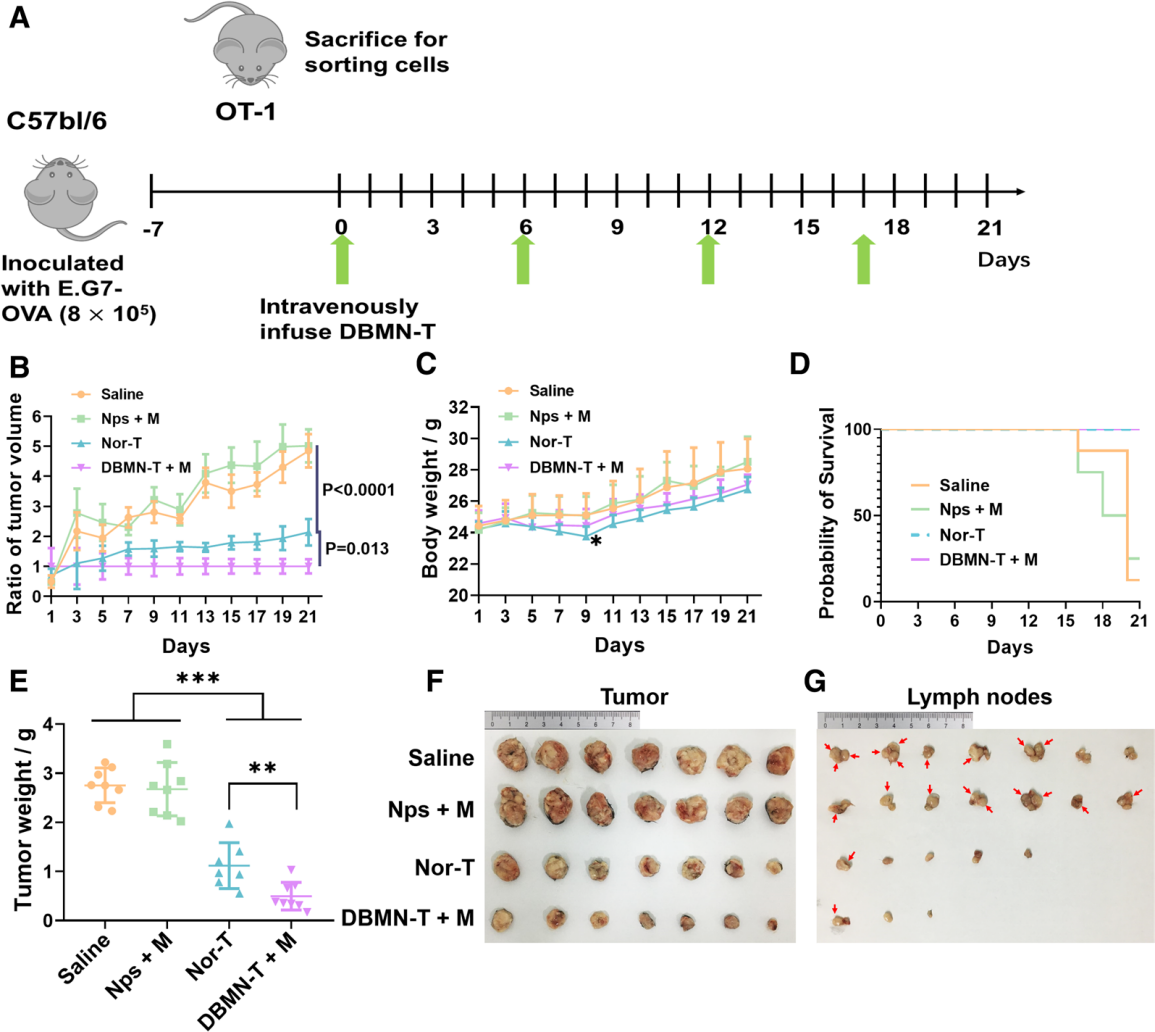
Anti-tumor efficacy of T cells sorted from OT-I mouse in vivo. **A** Scheme of the anti-tumor experiment. **B** Tumor relative growth curves of mice in different groups, n = 7–9. Changes in body weight (**C**) and survival rate (**D**) of mice during treatment. Mice with tumors larger than 2000 mm^3^ were dead by default. The actual weight (**E**) and photos (**F**) of tumors at day 21. **G** Metastasis of tumor cells in lymph nodes. Red arrows, obvious metastases sites. Statistical analyses were carried out by Prism graphpad 8.0. All error bars are expressed as ± SD. Comparisons between two or several groups were analyzed using unpaired Student’s t-test, *p < 0.05, **p < 0.01, ***p < 0.001, ****p < 0.0001

CD8 + T cells from OT-I were negatively sorted by mouse CD8 + T cell negative enrichment kit to specifically recognize E.G7-OVA tumor cells. The tumor volumes in the group treated with T cells were significantly smaller than that of the saline and nanoparticles (Nps + M) groups (Fig. 4A). Meanwhile, the tumors in the DBMN-T group were smallest, demonstrating a distinct inhibitory effect (decreased by 105%) on solid tumors compared with normal adoptive T cell (Nor-T) therapy (Fig. 4B, C, E, F), with a significantly prolonged survival time of mice (Fig. 4D). It was reported that E.G7-OVA tumors generally had a high tendency to metastasize toward lymph nodes[39]. And the interference of adoptive CD8 + T cells dramatically suppressed the metastasis of cancer cells, especially in the DBMN-T group (Fig. 4G).

**Fig. 5:**
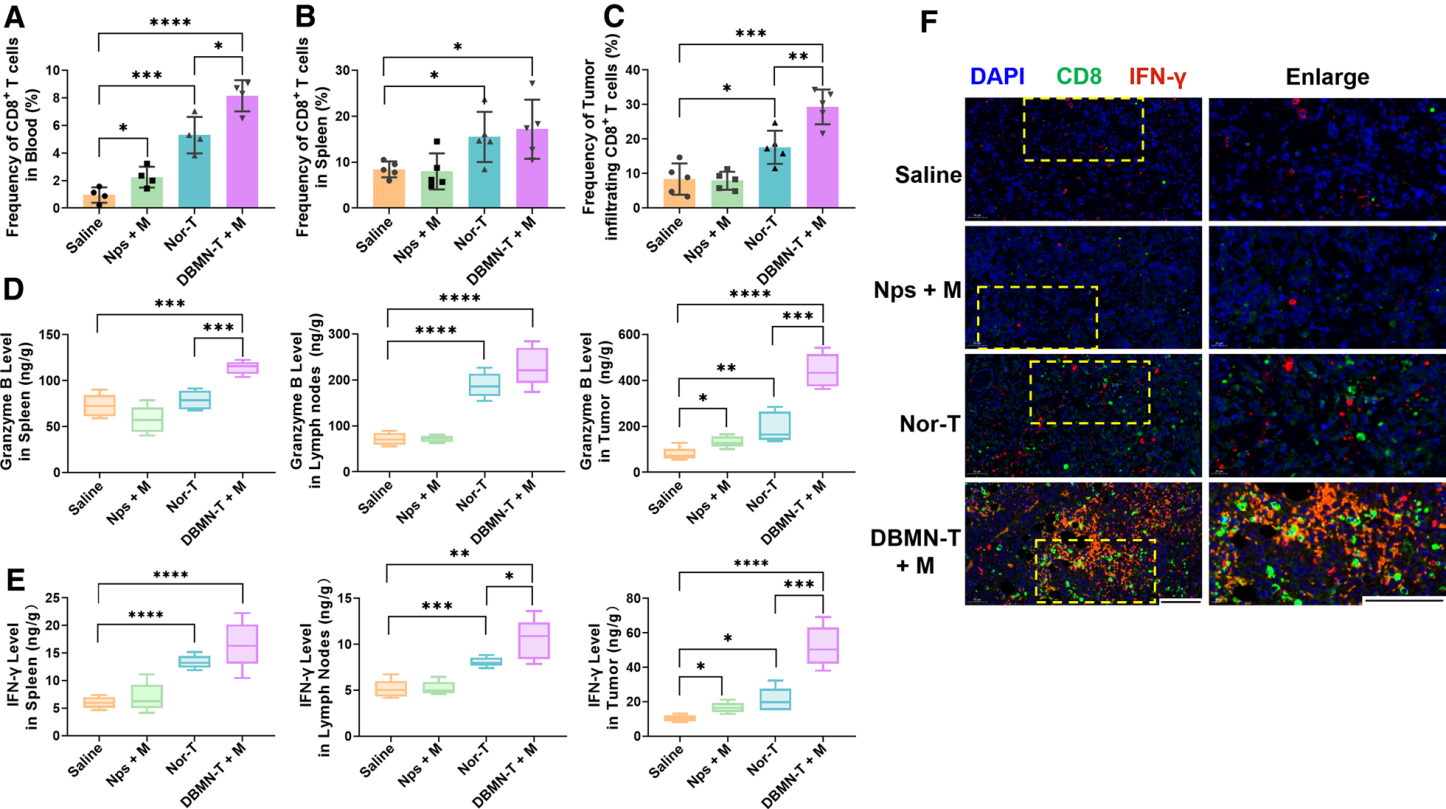
Immune responses based on OT-I T cells reinfusion therapy. Flow cytometry analysis of CD8 + T cells proportion in blood (**A**), spleens (**B**) and tumors (**C**) by Flowjo 10, n = 5. Granzyme B (**D**) and IFN-γ (**E**) content in spleens, lymph nodes and tumors detected by Elisa assay, n = 5. **F** Immunofluorescence images of CD8 + T cells and IFN-γ in tumor tissues. Scale bar, 100 μm. Statistical analyses were carried out by Prism graphpad 8.0. All error bars are expressed as ± SD. Comparisons between two or several groups were analyzed using unpaired Student’s t-test, *p < 0.05, **p < 0.01, ***p < 0.001, ****p < 0.0001

At the end of the experiment, mice were sacrificed with major organs and tumors collected for further analysis. In the blood and tumors, there was a remarkable increase in CD8 + T cells in DBMN-T group compared with other groups as determined by flow cytometry. The proportion of CD8 + T cells were elevated after ACT (Fig. 5A–C, Additional file 1: Fig. S10). Moreover, this percent in tumors of DBMN-T group (Vs normal T cells group) increased by 75%. The level of cytokines in the spleens, lymph nodes and tumors were tested by Elisa assay. Compared with reinfusion of normal T cell, the concentrations of IFN-γ and granzyme B were higher in the DBMN-T group. And the biggest disparity appeared in tumors, with the upregulation to 227% in granzyme B and 248% in IFN-γ (Fig. 5D, E). The literature showed that cytokine levels were closely related to cytokine storm [40]. Thus, we introduced safety factors (SF_IFN−γ_ = Spleen IFN-γ level/Tumor IFN-γ level + Lymph nodes IFN-γ level/Tumor IFN-γ level) to evaluate the safety of ACT. A smaller value of the SF confirmed higher relative biosafety. The SF_IFN−γ_ and SF_GzmB_ of DBMN-T were 0.52 and 0.78, respectively. While these were 1.02 and 1.37 in normal T cells group, approximately twice the value of the new treatment. By adoptive DBMN-T cell therapy, cytokines relatively enriched in the tumor sites, which reversed the immunosuppressive TME. Simultaneously, our strategy successfully guarded against the cytokine storm. Immunofluorescence images intuitively reproduced the above phenomenon (Fig. 5 F, Additional file 1: Fig. S11). Furthermore, the mice in normal T cell group experienced severe weight loss after reinfusion (day 3 to 9, p < 0.05), while the symptom in DBMN-T group was mild (Fig. 4C). More safety evaluations were reflected in H&E staining. Mice infused with CD8 + T cells had little metastases and inflammation, while the mice in other groups endured malignant metastases especially in the liver and kidney (Additional file 1: Fig. S12).

### The dual targeting of DBMN

**Fig. 6:**
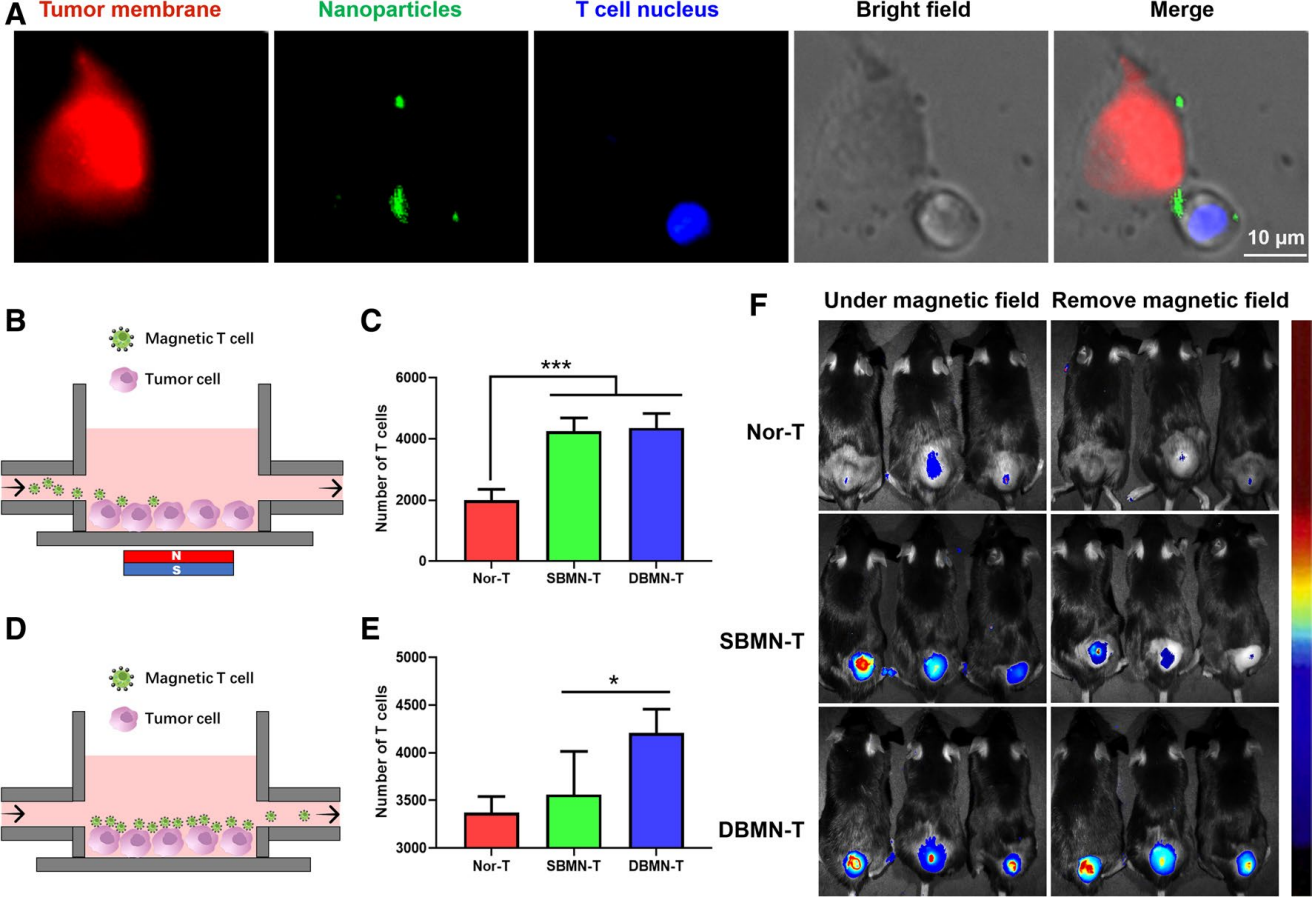
The DBMN helped T cells adhere to tumor cells. **A** The CD8 + T cell–Nanoparticle–Tumor cell bridge was built when DBMN-T were cultured with 4T1 tumor cells. Red, DiD-labeled 4T1 cell membrane; Green, FITC-DBMN; Blue, Hoechst labeled CD8 + T cell Nucleus. Scale bar, 10 μm. **B** Pattern diagram of adhesion test. Circulating T cells attracted under a magnetic field and the number of CD8 + T cells (**C**) adhered to 4T1 cells counted by Image J, n = 4. **D** Pattern diagram of rinsing test. Free medium rinsed CD8 + T cells attached on 4T1 cells and the number of remaining CD8 + T cells (**E**) counted by Image J, n = 4. **F** Fluorescence images of normal T cells, SBMN-T and DBMN-T, respectively. Left column, under magnetic field for 48 h; Right column, without magnetic field for 24 h after previous step. Statistical analyses were carried out by Prism graphpad 8.0. All error bars are expressed as ± SD. Comparisons between two or several groups were analyzed using unpaired Student’s t-test, *p < 0.05, **p < 0.01, ***p < 0.001

Apart from the magnetic field, we believed that there were definitely other factors that assist in the residence of T cells at the tumor sites. We assumed that DBMN, as an intermediate, built a T cell–nanoparticle–tumor cell bridge (Fig. 6A), linking T cells and tumor cells synchronously in TME. CD44 was highly expressed on specific tumor cells (Additional file 1: Fig. S13). And HA as the vital part of DBMN is a natural ligand of CD44, playing a key role in enriching T cells around tumor cells. To further verify this, we synthesized single-binding magnetic nanoparticles (SBMN, size = 242 ± 31 nm, ζ potential = 7.2 ± 3.1 mV) without HA. The responsiveness of DBMN-T and SBMN-T were determined by adhesion test (Fig. 6B). Circulating magnetic T cells were captured in a petri dish under magnetic field, results in Fig. 6C meant similar magnetic responsiveness of SBMN and DBMN modified CD8 + T cells. On this basis, rinsing test was performed according to the diagram shown in Fig. 6D. CD8 + T cells were co-incubated with 4T1 tumor cells in advance. In order to simulate the effect of blood flow in the body, free medium was used to rinse CD8 + T cells attached on the tumor cells. Excitingly, DBMN modified T cells showed a stronger adhering capacity to tumor cells (Fig. 6E). The biodistribution also confirmed an analogous effect. Under magnetic field, we reinfused DiR-labeled T cells (containing Nor-T, DBMN-T and SBMN-T, respectively). 48 h later, DBMN-T and SBMN-T were attracted into the tumor sites compared to normal T cells (Fig. 6F). Next, magnets were removed and the fluorescence was affirmed again. Interestingly, the relative fluorescence intensity of SBMN-T sharply decreased by 27% compared with DBMN-T 24 h after removing the magnetic field. These evidences proved the enrichment capacity of CD8 + T cells was vitally related to the DBMN.

## Conclusion and discussion

Although genetically engineered T cell therapy is recognized as one of the most promising immunotherapies, it encountered many barriers in treating non-hematological malignancies, especially the poor accessibility and infiltration toward solid tumor, which hinders the future development and clinical application of CAR-T therapy.

Previous approaches mainly focused on increasing the ability of CTL to kill tumor cells by developing stronger CAR-T cells. It was a traditional method by eliminating malignancy from outside to inside which still cannot solve the problem of less T cell infiltration in tumor.

Magnetic targeting is the most effective external control targeting method, which can precisely control the movement of drugs, nanocarriers and cells in the body. Here, we designed a magnetic nanoparticle with multifaceted tumor-targeting properties to directly increase T cells invasion in tumors as Scheme 1. By reacting with maleimide, magnetic T cells (DBMN-T) were obtained and its magnetic responsiveness in vitro (Fig. 1D, F, Additional file 2: Movie S1, Additional file 3: Movie S2) and in vivo (Figs. 3 and 6F) were verified. Meanwhile, the modification of nanoparticles did not affect the function of T cells, especially for the secretion of cytokines, like IFN-γ and granzyme B (Fig. 2). Then, in the mice subcutaneous E.G7-OVA tumor model, DBMN-T showed best anti-tumor effect (Fig. 4B, E, F) with fewer side effects (Fig. 4C) which may contributed to more centralized adoptive T cells at tumor sites. Next, we realized that DBMN-T could adhere to the surface of tumor highly expressed CD44, enhance and maintain targeting efficacy (Fig. 6). In this case, DBMN played a bridging role in connecting T cell and tumor cell, which was adopted in the latest studies seem to strengthen the inhibitory ability of T cells [28, 29], demonstrating a superior tumor-specific accumulation and effector killing.

What’s more, this was a different direction than developing stronger CAR-T cells. A simple mixing with immune cells (half an hour) can complete manufacturing, which may be added in treatment flow-sheet, improving the effect of solid tumors simultaneously from different perspectives.

## Methods

### Reagents

Magnetic iron oxide nanoparticles modified with amino groups (PuriMag G-NH_2_ 200 nm, MN) were purchased from PuriMag Biotechnology Ltd, (Xiamen, China). Hyaluronic acid (HA, molecular weight: ~ 5700 Da) was obtained from Freda Biochemical Co., Ltd, (Shandong, China). NH_2_-PEG_1000_-Mal was synthesized by ToyongBio Tech Ins, (Shanghai, China). 1-Ethyl-3 (3-dimethylaminopropyl) carbodiimide (EDC), N-Hydroxysuccinimide (NHS) and 4-Maleimidobutyric Acid (MBA) were purchased from Aladdin Biochemical Technology Co., Ltd, (Shanghai, China). 1,1’-Dioctadecyl-3,3,3’,3’-Tetramethylindodicarbocyanine Perchlorate (DiD) and 1,1’-dioctadecyltetramethyl indotricarbocyanine Iodide (DiR) were from Meilun biological company (Dalian, China). Other solvents were obtained from Sinopharm Chemical Reagent Co., Ltd. (Shanghai, China).

### Cell lines and culture conditions

Murine lymphoma E.G7-OVA cells genetically engineered to synthesize and secret OVA constitutively (C57BL/6 mice immunized with E.G7-OVA cells give rise to H-2K^b^ restricted CTLs specific for the OVA_258 − 276_ peptide) were obtained from BeNa Culture Collection (BNCC, Beijing, China). 4T1 cells and HEK293T-GFP cells (HEK293T cells encoding the GFP gene) were obtained from the Shanghai Cell Bank, Chinese Academy of Sciences. E.G7-OVA cells were cultured in Roswell Park Memorial Institute (RPMI) 1640 medium supplemented with 10% fetal bovine serum (FBS), 4T1 cells and HEK293T-GFP cells were cultured in Dulbecco’s modified Eagle medium (DMEM) containing 10% FBS. All cells maintained in cell incubator with 5% CO_2_.

### CD8^+^ T cells sorting and activation conditions

CD8 + T cells were obtained from C57BL/6 or OT-I mice (Slaccas Experimental Animal Co., Ltd. Shanghai, China). Briefly, mouse spleen was ground, and filtrated though a 200-mesh screen to obtain the single cell suspension, which was further sorted by mouse CD8 + T cell negative enrichment kit (ImunoSep, Beijing, China) to isolate and purify CD8 + T cells. The fresh CD8 + T cells (purity exceeded 95%) were cultured in T cell medium (RPMI 1640 supplemented with 10% FBS, 2 mM L-glutamine (Gibco, Thermo Fisher Scientific, USA), 1 mM sodium pyruvate (Gibco), 50 µM beta-mercaptoethanol (Gibco), 0.1 mM non-essential amino acids (Gibco) and 1 mM sodium pyruvate (Gibco). For T cell activation. a 5–10 µg/ml solution of anti-CD3e (145-2C11, BioLegend, California, USA) in sterile PBS was prepared and dispensed to 96-well plate (50 µL/well). After 2 hours’ incubation at 37 °C, each well was rinsed with sterile PBS to remove free (uncoated) antibodies. Then, CD8 + T cells were suspended in T-cell medium supplemented with 2 ug/mL of soluble anti-CD28 (37.51, BioLegend), 10 ng/mL of IL-2 (PeproTech, New Jersey, USA), and 2 ng/mL of IL-7 (PeproTech) were plated. Cell entered the rapid proliferation phase after being incubated for 2 days. To specifically activate OT-I CD8 + T cells, bone marrow dendritic cell (BMDCs)were isolated from OT-I mice and cultured for 5–7 days using an established protocol [41]. Following the treatment with OVA peptide (SIINFEKL), BMDCs were matured with OVA-specific antigen presentation on the cell surface, which were further incubated with T cells (BMDCs : T cells = 1 : 5) for another 48 h to prime cognate CD8 + T cells.

### Synthesis of HMN, SBMN and DBMN

6 mg HA was activated with EDC and NHS for 2 h, and then 200 µL MN (10 mg/mL) was added to allow the reaction overnight. The products were absorbed with a magnet and washed three times to get purer HMN. The synthesis of SBMN was consistent with that of HMN, with slight changes. Specifically, 2.5 mg MBA was stirred with EDC and NHS quickly for 2 h, after that, MN was put into reaction system and finally SBMN was collected. DBMN was synthesized from HMN that owns excessive carboxyl groups. Similarly, 1 mL HMN (1 mg/mL) mixed with EDC and NHS solution was reacted with 2.3 mg NH_2_-PEG_1000_-Mal to get DBMN (plus with 0.1 mg FITC-NH_2_ could obtain FITC-DBMN).

### Characterization methods

The mean droplet size and the zeta potential were measured by dynamic light scattering (DLS) with a Zetasizer (Nano-ZS90, Malvern, UK). UV-visible absorption spectrum was acquired by using UV-visible spectrophotometer (Cary 60 UV-Vis, Agilent Technologies, USA). Thermogravimetric analysis (TGA) and differential scanning calorimetry (DSC) were acquired by Synchronous Thermal Analysis System (Q600 SDT, TA Instruments). The morphology was observed by Transmission Electron Microscopy (JEOL JEM-1230 microscopes, Japan). Finally, the prepared samples were lyophilized for further characterization.

### Nanoparticle conjugation with cells

The conjugation of nanoparticles on the cells was mainly referred to an established protocol [35, 36]. In general, 1 mg nanoparticles and 1 million T cells were co-incubated in a hydrosulfuryl-free culture medium. Culture plate was shaken every 10 min for 0.5 h. Finally, sulfhydryl-PEG was added and DBMN-T were collected by magnetic rack and centrifuged to transfer in fresh T cell medium.

### Magnetic responsiveness of magnetic cells in vitro

Magnetic HEK293T-GFP cells or DiD-labeled CD8 + T cells were cycled with a peristaltic circulation pump. A magnet was fixed on one side of the catheter (Additional file 1: Fig. S7A). Here, inverted fluorescence microscope (ECLIPSE Ti, NIKON) was used to observe the aggregation of cells, with IVIS imaging system (IVIS Spectrum, PE) used to observe the fluorescence change of whole catheter. To monitor the movement state of the magnetic cells, 1 million DBMN-T were gently dropped to one side of the petri dish and the magnet was placed on the other side (Fig. 1E). The movement of the magnetic cells was recorded with microscopy.

### Cell viability assay

To measure the proliferation capacity of normal T cells and DBMN-T, cells was resuspended in PBS and labelled with 1 µL CFSE (0.2 mg/mL in DMSO) (BioLegend) for 15 min. Afterwards, CFSE-labeled cells washed with complete medium to remove any free dyes. Then, cells were further cultured for 24 and 72 h before being post to flow cytometry analysis.

To evaluate the viability of normal T cells and DBMN-T, cells were seeded into 96-well plates at a density of 1 × 10^6^ per well. After incubating for 24 h, 10 µL CCK-8 solution was added to each well and incubated with cells for another 2 h. OD value was detected by microplate reader under 450 nm (iMark Microplate Reader, BIO-RAD, USA).

To investigate the cytokine release and cytotoxicity of normal T cells and DBMN-T, 5 × 10^5^ E.G7-OVA cells and 1 × 10^7^ OT-I T cells were seeded into 24-well plates successively. After 24 h, the expression of IFN-γ and granzyme B in the supernatant were measured by Elisa kits (Meimian Industrial Co., Ltd, Jiangsu, China). The release of lactate dehydrogenase (LDH) can be used to investigate the cell viability, thus, LDH release assay kit (Dojindo, Japan) was used to assay the killing tumor ability of normal T cells and DBMN-T.

To measure ATP production capacity of normal T cells and DBMN-T, cells were resuspended in fresh T cell medium, and ATP content was measured with ATP assay kit (Jiancheng Bioengineering Institute, Nanjing, China) according to the manufactures’ instructions.

### Animal models

6–8 weeks old female C57BL/6 or OT-I mice were purchased from Slaccas Experimental Animal Co., Ltd. (Shanghai, China) and used for all the following animal studies. All in vivo experiments were performed in compliance with the requirements of the Zhejiang University Animal Study Committee for the care and use of laboratory animals in research. E.G7-OVA tumor model was established by subcutaneously injecting 8 × 10^5^ E.G7-OVA cells into the back skin of mice. And for bilateral tumor, 5 × 10^5^ E.G7-OVA cells were injected on both sides of the back, respectively.

### 10 T cell enrichment in vivo

DiR-labeled DBMN-T was intravenously injected into mice with bilateral tumor. A magnet was fixed on the left tumor for 48 h. The fluorescence was observed under an IVIS system (PerkinElmer). Afterwards, mice were sacrificed with the tumors on both sides sectioned separately. Labeled CD8 + T cells were detected, and the iron oxide nanoparticles were stained with Prussian blue.

To detect the tumor enrichment ability of different T cells. DiR-labeled normal T cells, SBMN-T and DBMN-T were intravenously injected into mice bearing E.G7-OVA tumor on the back. The fluorescence was measured after 48 h under the magnetic field. Then, the magnet was removed and the fluorescence was measured again after another 24 h.

### Anti-tumor efficacy in vivo

E.G7-OVA tumor bearing C57/BL6 mice were randomly divided into 4 groups: Saline (inject saline every other week), Nps + M (nanoparticles + M, inject 0.5 mg nanoparticles every other week), Nor-T (normal T cells, inject 8 million normal T cells every other week) and DBMN-T + M (induce 8 million DBMN-T every other week). For groups with M, mice were placed under the magnetic field for 48 h after injection. The body weight and tumor volume were recorded every other day (tumor volume = length × width × height/2). Mice were sacrificed at the end of the experiment. Blood, spleen, lymph nodes and tumor tissues were collected for flow cytometry analysis and Elisa analysis, while the main organs (heart, liver, spleen, lung, kidney) were collected for hematoxylin-eosin staining.

### ELISA assay

About 200 mg tumor, whole spleen and whole lymph node were weighed for further experiments and the exact weight of organs was recorded. Then, after cutting specimens, cell lysate was obtained by mini Cell High Speed Shearer (10,000 rpm for 3 min) in 1 mL cold PBS and centrifuged at 30 rpm for 20 min. The supernatant (50 µL) was collected for ELISA assay. The operation steps of ELISA assay were mainly in accordance with the manufacturer’s instructions. In brief, the samples were added in ELISA wells and incubated for 30 min at 37 ℃. Then, discard liquid, add washing buffer to each well, repeat 5 times. Next, HRP-Conjugate reagent was added to the wells and incubated for 30 min at 37 ℃, washed again. Next, Chromogen Solution A and B were added and incubated for 15 min at 37 ℃ before adding Stop Solution. The absorbance of each well was measured at 450 nm by Microplate reader. Specific concentrations of cytokines were standardized by standard curves and exact weight.

### Adhesion test and rinsing test

The culture plate was installed with water inlet and outlet and outlet on two sides. In addition, a peristaltic pump was installed to circulate. For adhesion test, 4T1 cells were seeded into plates for 12 h to allow cells attached to the plate bottom. Hoechst-labeled normal T cells, SBMN-T and DBMN-T were circulated with culture medium flowing through plate for 5 min, respectively. The blue fluorescence was measured by inverted fluorescence microscope. For rinsing test, 4T1 cells were seeded into plates to allow attached to the plate bottom. Next, normal T cells, SBMN-T and DBMN-T (T cells : tumor cells = 20 : 1) were added to adhere to 4T1 cells for 4 h. To simulate the flushing of blood flow, cells were rinsed by fresh medium for 5 min. The fluorescence images were photographed and cell number was counted by Image J.

## Statistical analysis

Data were represented as mean ± standard error. Comparisons between two or several groups were analyzed using unpaired Student’s t-test or one-way analysis of variance (ANOVA) by Newman–Keuls test. All statistical analyses were carried out by Prism-GraphPad version 8.0 (San Diego, CA), with a value of p < 0.05 considered to be statistically significant.

## Supplementary Information

**Additional file 1: Figure S1.** The structure of DBMN. **Figure S2. **Size (A) and zeta potential (B) distributionof MN, HMN and DBMN tested by DLS.** Figure S3. TGA (A) and DSC (B) curves of MN, HMN andDBMN. C) The image of the samples after being subjected to a high temperatureof 1000 °C. Figure S4. **UV-Vis absorption spectra of the mainreactants and products. **Figure S5. **MN and DBMN accumulated over time under the magnetic field.** Figure S6. **Magnetic responsiveness of T cells after incubatingwith DBMN. Culture plate was shaken every 10 minutes for 0.5 hours.** Figure S7. **A)The scheme of circulation of DBMN-HEK293T-GFP cells in vitro under the magnetic field. B) Fluorescence image ofcatheter near the magnet (This image was composed of three pictures from top tobottom).** Figure S8. **The photos of thedevice for verifying the magnetic responsiveness of magnetic cells by IVIS. **Figure S9.**Fluorescence images of DBMN-HEK293T-GFP cells undermagnetic field, captured by IVIS. Blue dashed box, the magnet.** Figure S10.**Representative flow cytometry pictures of CD8+ T cells proportion inblood (A), spleens (B) and tumors (C).** Figure S11.**Representative immunofluorescence images of CD8^+^T cells and IFN-γ in DBMN-T group. Scale bar, 50 µm.** FigureS12.**Representative H&E staining photographs of hearts, livers, spleens,lungs and kidneys in each group of mice. Yellow arrow, tumor metastasis site. Scale bar,100 µm. **Figure S13. **The expression of CD44 on 4T1 cells and E.G7-OVA cells. Left is blank.

**Additional file 2: Movie S1.** Magnetic T cells moved quickly under the magnetic field.

**Additional file 3: Movie S2.** Magnetic T cell adjusted its direction as the change of the magnetic field.

## Data Availability

The data and materials of the study can be obtained from the corresponding author upon request.
