# Supplementary material for: Dual-binding nanoparticles improve the killing effect of T cells on solid tumor
Source: J Nanobiotechnology. 2022 Jun 7;20:261. doi: 10.1186/s12951-022-01480-z (PMC9171930; doi:10.1186/s12951-022-01480-z)
Supplement: Supplementary file 1 — Additional file 1: Figure S1. The structure of DBMN. Figure S2. Size (A) and zeta potential (B) distributionof MN, HMN and DBMN tested by DLS. Figure S3. TGA (A) and DSC (B) curves of MN, HMN andDBMN. C) The image of the samples after being subjected to a high temperatureof 1000 °C. Figure S4. UV-Vis absorption spectra of the mainreactants and products. Figure S5. MN and DBMN accumulated over time under the magnetic field. Figure S6. Magnetic responsiveness of T cells after incubatingwith DBMN. Culture plate was shaken every 10 minutes for 0.5 hours. Figure S7. A)The scheme of circulation of DBMN-HEK293T-GFP cells in vitro under the magnetic field. B) Fluorescence image ofcatheter near the magnet (This image was composed of three pictures from top tobottom). Figure S8. The photos of thedevice for verifying the magnetic responsiveness of magnetic cells by IVIS. Figure S9.Fluorescence images of DBMN-HEK293T-GFP cells undermagnetic field, captured by IVIS. Blue dashed box, the magnet. Figure S10.Representative flow cytometry pictures of CD8+ T cells proportion inblood (A), spleens (B) and tumors (C). Figure S11.Representative immunofluorescence images of CD8+T cells and IFN-γ in DBMN-T group. Scale bar, 50 µm. FigureS12.Representative H&E staining photographs of hearts, livers, spleens,lungs and kidneys in each group of mice. Yellow arrow, tumor metastasis site. Scale bar,100 µm. Figure S13. The expression of CD44 on 4T1 cells and E.G7-OVA cells. Left is blank. [file 12951_2022_1480_MOESM1_ESM.docx]

**Supporting information:**

**Dual-binding nanoparticles improve the killing effect of T cells on solid tumor**

*Zhenyu Luo^1^, Lihua Luo^1^, Yichao Lu^1^, Chunqi Zhu^1^, Bing Qin^1^, Mengshi Jiang^1^, Xiang Li^1^, Yingying Shi^1^, Junlei Zhang^1^, Yu Liu^1^, Xinyu Shan^1^, Hang Yin^1^, Guannan Guan^1^, Yongzhong Du^1^, Ningtao Cheng^2^*, Jian You^1^**

Authors’ Affiliations:

1 College of Pharmaceutical Sciences, Zhejiang University, 866 Yuhangtang Road, Hangzhou, Zhejiang 310058, P. R. China

2 School of Public Health, Zhejiang University School of Medicine, 866 Yuhangtang Road, Hangzhou, Zhejiang 310058, P. R. China

* Corresponding Author:

Jian You, College of Pharmaceutical Sciences, Zhejiang University, 866 Yuhangtang Road, Hangzhou, Zhejiang 310058, P. R. China. Email: [youjiandoc@zju.edu.cn](mailto:youjiandoc@zju.edu.cn).

Ningtao Cheng, School of Public Health, Zhejiang University School of Medicine, 866 Yuhangtang Road, Hangzhou, Zhejiang 310058, P. R. China. Email: [ncheng@zju.edu.cn](mailto:ncheng@zju.edu.cn)


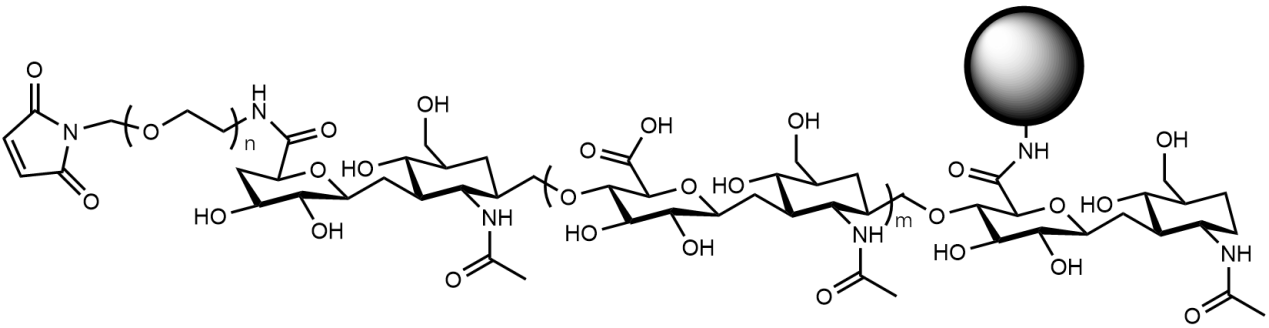


Figure S1. The structure of DBMN.


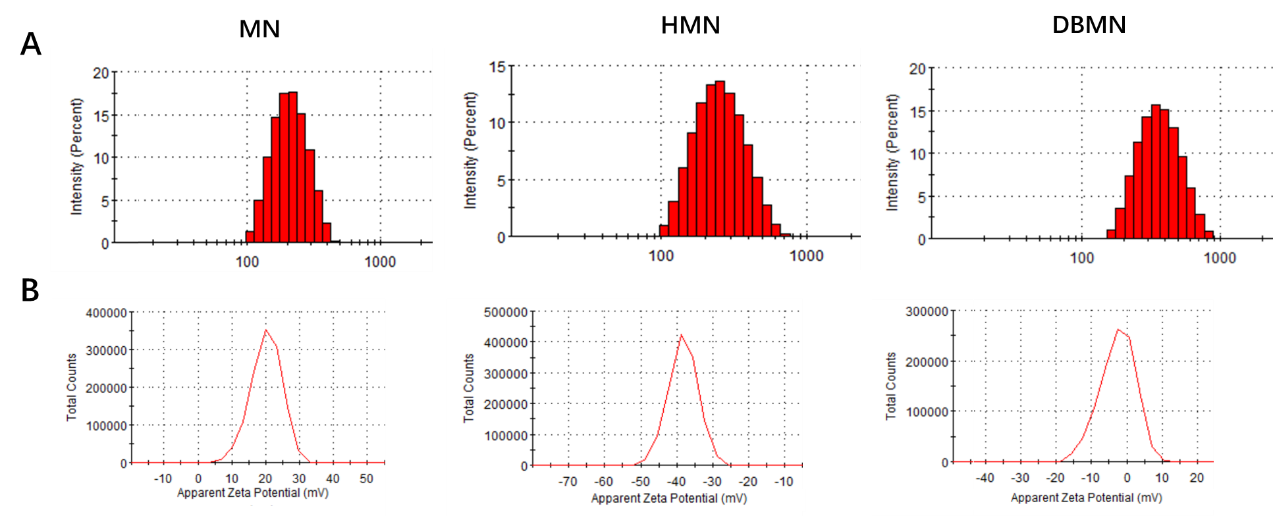


Figure S2. Size (A) and zeta potential (B) distribution of MN, HMN and DBMN tested by DLS.


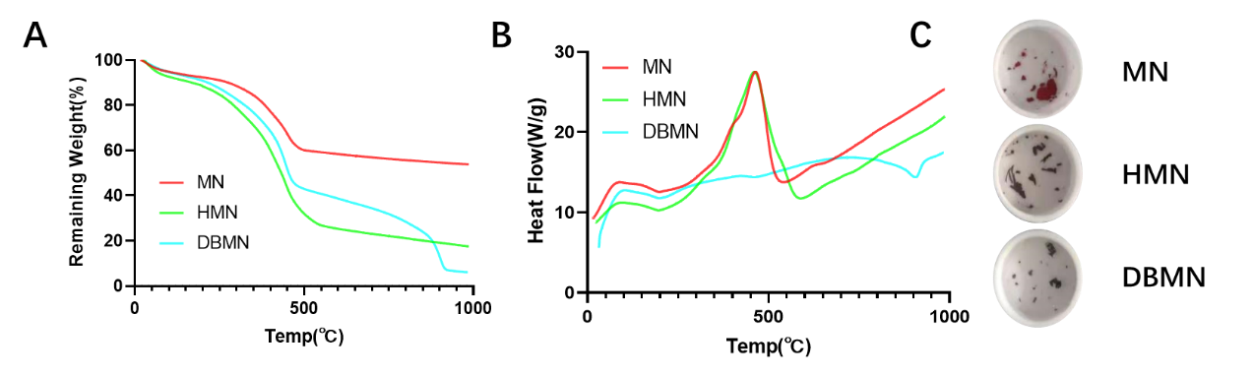


Figure S3. TGA (A) and DSC (B) curves of MN, HMN and DBMN. C) The image of the samples after being subjected to a high temperature of 1000 °C.

Figure S4. UV-Vis absorption spectra of the main reactants and products.


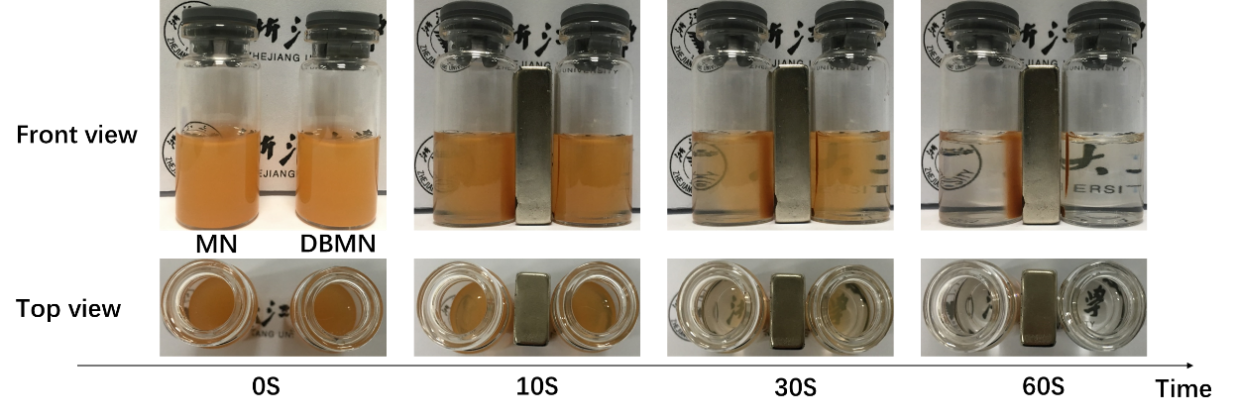


Figure S5. MN and DBMN accumulated over time under the magnetic field.

Figure S6. Magnetic responsiveness of T cells after incubating with DBMN. Culture plate was shaken every 10 minutes for 0.5 hours.


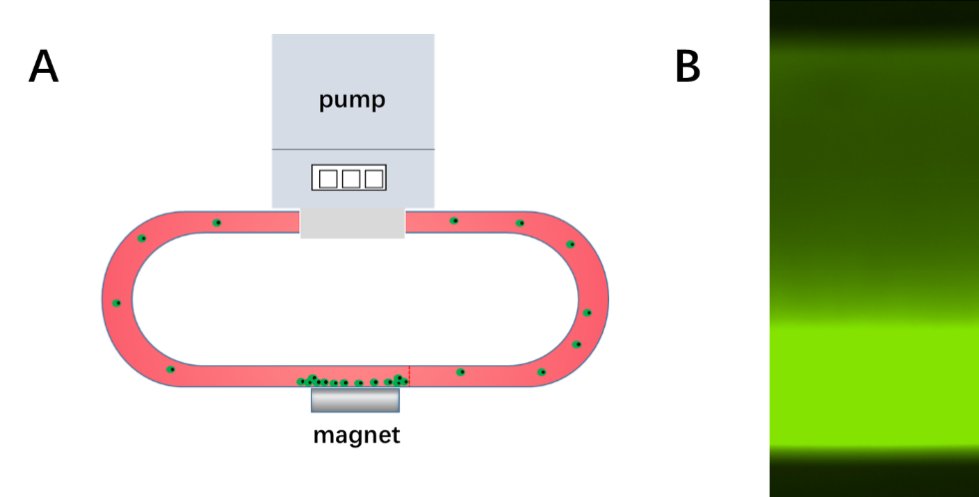


Figure S7. A) The scheme of circulation of DBMN-HEK293T-GFP cells *in vitro* under the magnetic field. B) Fluorescence image of catheter near the magnet (This image was composed of three pictures from top to bottom).


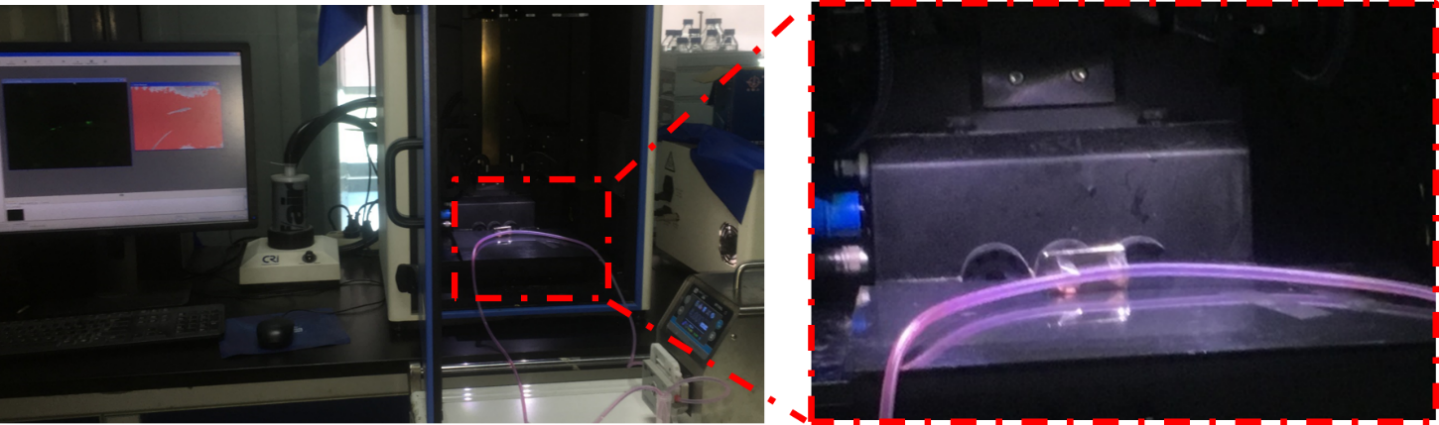


Figure S8. The photos of the device for verifying the magnetic responsiveness of magnetic cells by IVIS.


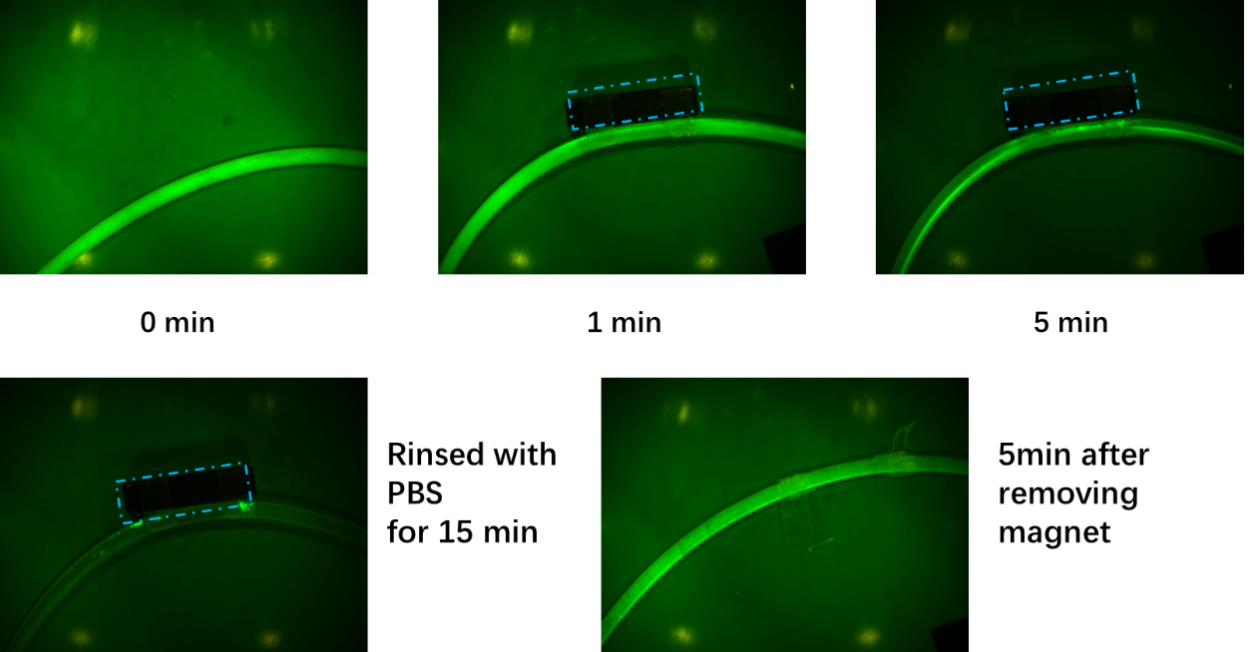


Figure S9. Fluorescence images of DBMN-HEK293T-GFP cells under magnetic field, captured by IVIS. Blue dashed box, the magnet.


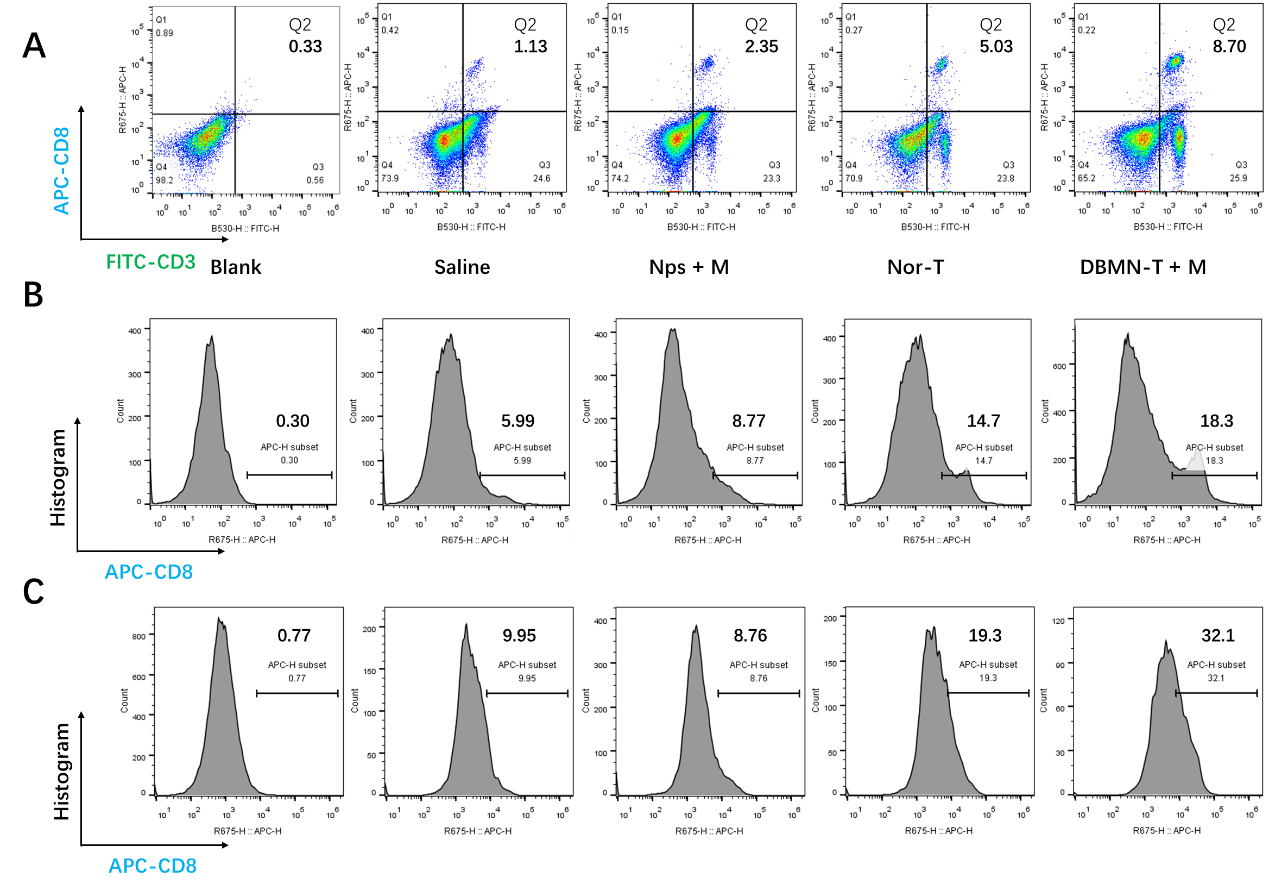


Figure S10. Representative flow cytometry pictures of CD8+ T cells proportion in blood (A), spleens (B) and tumors (C).


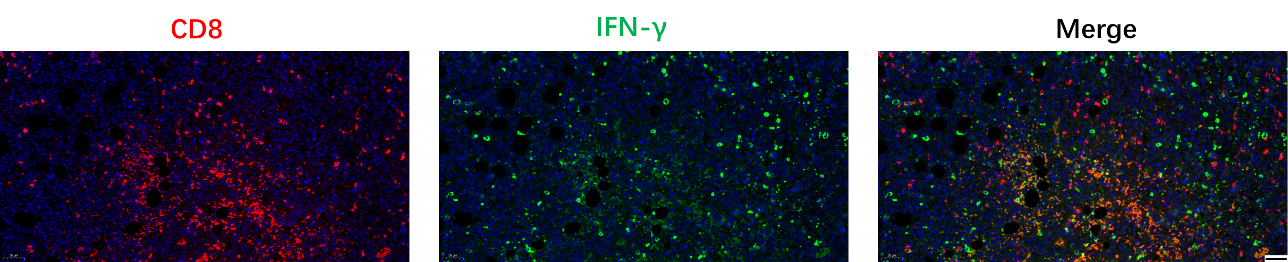


Figure S11. Representative immunofluorescence images of CD8^+^ T cells and IFN-γ in DBMN-T group. Scale bar, 50 µm.


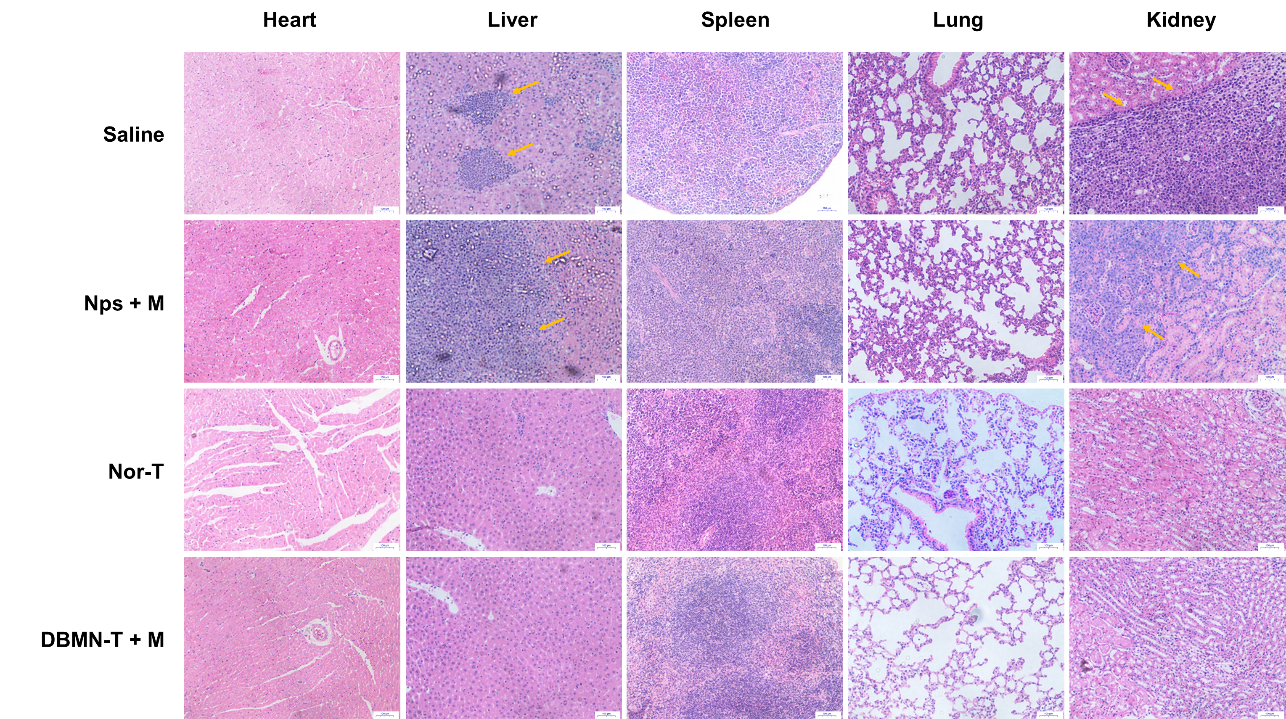


Figure S12. Representative H&E staining photographs of hearts, livers, spleens, lungs and kidneys in each group of mice. Yellow arrow, tumor metastasis site. Scale bar, 100 µm.


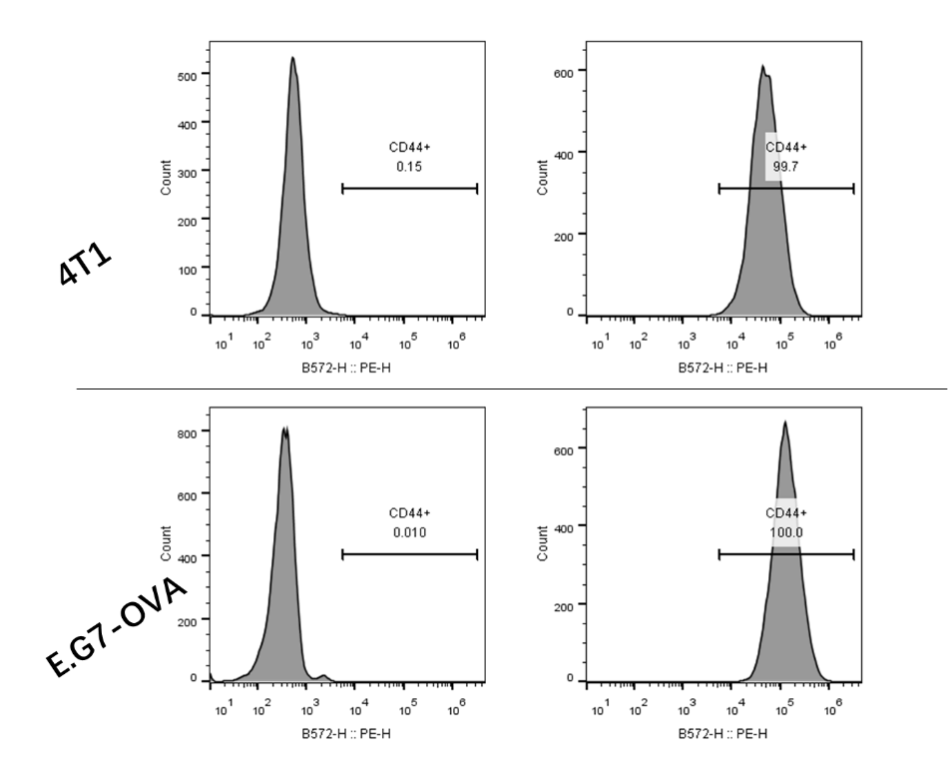


Figure S13. The expression of CD44 on 4T1 cells and E.G7-OVA cells. Left is blank.
